# Can ^129^I track ^135^Cs, ^236^U, ^239^Pu, and ^240^Pu apart from ^131^I in soil samples from Fukushima Prefecture, Japan?

**DOI:** 10.1038/s41598-017-15714-w

**Published:** 2017-11-13

**Authors:** Guosheng Yang, Hirofumi Tazoe, Masatoshi Yamada

**Affiliations:** 1 Department of Radiation Chemistry, Institute of Radiation Emergency Medicine, Hirosaki University, 66-1 Hon-cho, Hirosaki, Aomori, 036-8564 Japan; 2 Division of Nuclear Technology and Applications, Institute of High Energy Physics, Chinese Academy of Sciences, Beijing, China; 3 Beijing Engineering Research Center of Radiographic Techniques and Equipment, Beijing, 100049 China

## Abstract

In the present study, ^129^I activities and ^129^I/^127^I atom ratios were measured in 60 soil samples contaminated by the Fukushima Daiichi Nuclear Power Plant (FDNPP) accident. The ^127^I concentrations, ^129^I activities, and ^129^I/^127^I atom ratios in dry-weight were observed to be 0.121–23.6 mg kg^−1^, 0.962–275 mBq kg^−1^, and (0.215–79.3) × 10^−7^, respectively. The maximum values of both ^129^I activities and ^129^I/^127^I atom ratios in Japanese soil increased about three orders of magnitude due to this accident. The equation logy = 0.877logx + 0.173 (Pearson’s r = 0.936; x, ^129^I concentration; y, ^131^I concentration; decay-corrected to March 11, 2011) instead of a simple constant may be a better way to express the relationship between ^129^I and ^131^I in Japanese soil affected by both global fallout and FDNPP accident fallout. In addition, a moderate correlation was observed between ^129^I and ^135^Cs (logy = 0.624logx + 1.01, Pearson’s r = 0.627; x, ^129^I activity; y, ^135^Cs activity). However, ^129^I presented larger fractionations with less volatile radionuclides, such as ^236^U, ^239^Pu, and ^240^Pu. These findings indicated ^135^Cs could be roughly estimated from ^129^I or ^131^I; this is advantageous as fewer ^135^Cs data are available and ^135^Cs/^137^Cs is being considered a promising tracer during radiocesium source identification.

## Introduction

The Fukushima Daiichi Nuclear Power Plant (FDNPP) accident in 2011 released massive amounts of radionuclides into the terrestrial environment, including both short-lived radionuclides (e.g. ^133^Xe, 5.2 d; ^131^I, 8.0 d; ^133^I, 20.8 h; ^134^Cs, 2.1 y) and long-lived radionuclides (e.g. ^129^I, 1.57 × 10^7^ y; ^135^Cs, 2.3 × 10^6^ y; ^236^U, 2.342 × 10^7^ y; ^239^Pu, 24,110 y and ^240^Pu, 6,564 y)^1,2^. The radionuclides with shorter half-lives have higher specific radioactivities, making them suitable for conventional radiometric methods^3^. Furthermore, conventional radiometric methods, such as γ spectrometry, are easier to apply as they do not have complicated procedures for sample treatment, chemical separation, and purification. Therefore, those short-lived radionuclides with high radiation exposure risk, such as ^131^I and ^134^Cs, were extensively studied in the initial stage of the FDNPP accident. On the other hand, the data for long-lived radionuclides, such as ^129^I, ^135^Cs, ^236^U, ^239^Pu, and ^240^Pu related to the FDNPP accident are limited^4^.

Investigation of the distributions and their relevance of ^129^I, ^135^Cs, ^236^U, ^239^Pu, and ^240^Pu in Japanese soil is highly required for three major reasons. First, ^129^I, ^135^Cs, ^236^U, and ^239+240^Pu were indeed released from the FDNPP accident, and their amounts were estimated to be (0.66–5.5) × 10^10^, 6.74 × 10^10^, 1.2 × 10^6^, 2.3 × 10^9^ Bq, respectively^1,5,6^. Second, these long-lived radionuclides can be determined in environmental samples for a long time after a nuclear accident; therefore, they have great potential to act as proxies for short-lived radionuclides that are of greater radiological concern. Initial reconstructions of the distribution of ^131^I deposition through the measurement of ^129^I were obtained, as it was known that there were strong correlations between ^131^I and ^129^I activities in the contaminated surface soil samples affected by both the Chernobyl accident^7^ and the FDNPP accident^8–11^. Third, they are well-suited tracers with great potential for source identification. ^135^Cs/^137^Cs has been proved to be a powerful alternative tracer of ^134^Cs/^137^Cs for radiocesium source identification to overcome the drawback of the short half-life of ^134^Cs in studies of the FDNPP accident^5,12–14^. Significant work has been done that revealed the release of trace amounts of Pu during the FDNPP accident and proved ^240^Pu/^239^Pu is a good tracer for Pu source identification^15–17^. At the same time, many scientists have been trying to find evidence of ^236^U release during the FDNPP accident^6,18–21^. Finally, Shinonaga *et al*.^19^ and Yang *et al*.^21^ were able to present evidence of ^236^U release from this accident in aerosol samples and soil samples, respectively.

Different elements have distinct physicochemical properties, which will result in different dispersal and deposition processes from reactor cores to the environment at the initial stage of their emissions and different post-depositional redistributions (vertical diffusion or migration) in soil samples. For example, evidence was observed that ^129^I migrated downward more rapidly in soil than ^137^Cs did after both the Chernobyl accident^22^ and the FDNPP accident^23^. Therefore, it is better to apply a long-lived isotope to act as a proxy for a short-lived isotope of the same element, such as ^129^I for ^131^I, and ^135^Cs for ^134^Cs and ^137^Cs. However, it should be noted that only trace amounts of ^129^I, ^135^Cs, ^236^U, ^239^Pu and ^240^Pu were released from the FDNPP accident, and background activities before this accident account for the major fraction of these radionuclides, as revealed by previous studies and mentioned above. For example, before the FDNPP accident, ^129^I was already present in Japanese soil owing to natural generation (cosmic ray reactions with Xe, spontaneous fission of ^238^U, thermal neutron induced fission of ^235^U and Te) and human nuclear activities (atmospheric nuclear test since 1945, discharge from spent-nuclear-fuel reprocessing plants, fallout from the Chernobyl accident). Multiple sources and long-term redistributions make their relationships complicated. However, most reports only focused on one or two long-lived radionuclides in each study. Therefore, it is necessary to build a database to show data for as many as possible long-lived radionuclides in the same samples to establish their signatures and illustrate the differences and relevance among them in soil samples affected by the FDNPP accident fallout and global fallout.

Unfortunately, there are still not sufficient data related to the FDNPP accident for environmental samples from Fukushima Prefecture to provide regional information on the deposition of multiple long-lived radionuclides, such as, ^129^I, ^135^Cs, ^236^U, ^239^Pu and ^240^Pu. The major reason for the limited numbers of data of long-lived radionuclides is the challenge in their measurement. Since mass spectrometric methods are more sensitive for longer-lived radionuclides^3^, inductively coupled plasma - mass spectrometry (ICP-MS) is the most widely applied method to measure Pu isotopes^15–17^. For ^129^I and ^236^U in environmental samples, accelerator mass spectrometry (AMS) is the most widely applied and it offers the highest sensitivities^6,18–21,24–26^. However, due to the high instrument cost, there are only about 110 AMS facilities worldwide, and most of them are mainly applied to the routine analysis of ^14^C for dating purposes; only about ten can be used to study ^236^U^20,21,25^ and only about 22 AMS facilities can be used to study ^129^I^25^. Therefore, we have developed novel methods for rapid measurement of ^135^Cs, ^129^I and ^236^U in environmental samples with high throughput that are compatible with the advanced triple-quadrupole ICP-MS (ICP-QQQ)^14,20^. Compared with AMS, ICP-QQQ instruments are relatively inexpensive and can be afforded by ordinary laboratories, giving ICP-QQQ bright prospects in the field of long-lived ^135^Cs, ^129^I and ^236^U applications in the future.

Here, we report data for ^127^I, ^129^I, and ^131^I in 60 soil samples, with heavy ^134^Cs contamination due to the FDNPP accident, that were collected immediately after this accident. For those soil samples without ^131^I activity data, ^131^I activities were reconstructed via deduced ^129^I-^131^I equation and measured ^129^I activities. In addition, other radionuclides (major long-lived ones), such as ^135^Cs, ^236^U, ^239^Pu, ^240^Pu, were also considered with regard to the differences and relevance among them in soil samples affected by the FDNPP accident fallout and global fallout, in order to see whether ^129^I can track other radionuclides (^135^Cs, ^236^U, ^239^Pu, and ^240^Pu) derived from the FDNPP accident fallout and global fallout.

## Results

These 60 soil samples were collected in Fukushima Prefecture and were heavily contaminated with ^134^Cs (12.9–1.10 × 10^5^ Bq kg^−1^). Since ^134^Cs (t_1/2_ = 2.06 y) in the environment before the FDNPP accident has decayed out to an undetectable level^5,14,27^, these high values indicated significant radiocesium contamination due to the FDNPP accident. As shown in Supplementary Table S1, the ^127^I concentrations, ^129^I activities, and ^129^I/^127^I atom ratios in dry-weight were observed to be 0.121–23.6 mg kg^−1^, 0.962–275 mBq kg^−1^, and (0.215–79.3) × 10^−7^, respectively. In addition, the activities of ^135^Cs, ^236^U, ^239^Pu, and ^240^Pu in soil were in the ranges of 4.55–376, 0.005–0.244, 4.26–227, and 2.76–144 mBq kg^−1^, respectively.

## Discussion

**Figure 1:**
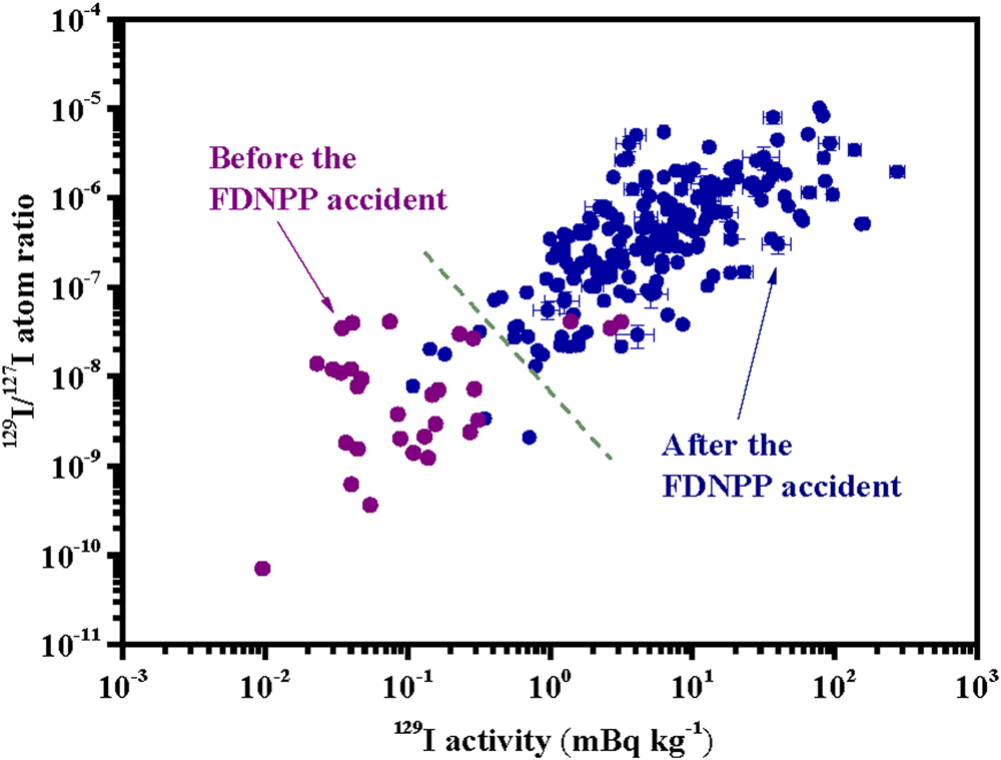
^129^I/^127^I atom ratio plotted against ^129^I activity in Japanese topsoil samples collected before and after the FDNPP accident. The dotted line mainly separated the data before and after the FDNPP accident into two cluster groups. The data were from our study and previous studies^8–11,29,30,35,36^ (the data from Miyake *et al*.^10^ and Muramatsu *et al*.^11^ did not show errors, other data were shown with errors of 1σ).

Presently, no reliable value is available for ^129^I/^127^I atom ratio in the terrestrial environment prior to the start of the nuclear era. Before the FDNPP accident, surface soil and atmospheric fallout collected far from the Tokai Reprocessing Plant (TRP) in Japan had ^127^I concentrations in somewhat over lapping ranges of 0.4–55.3^28–32^ and 2.79–110^33,34^ mg kg^−1^, respectively; the ^129^I activities were in the ranges of <0.06–4.55^28–32^ and 0.157–65.7^33,34^ mBq kg^−1^, respectively; and the ^129^I/^127^I atom ratios were relatively low, being in the ranges of (<0.1–2.18) × 10^−8^ ^28–32^ and (0.008–2.43) × 10^−7^ ^33,34^, respectively. For two surface soil samples collected in 2008 and 2009 within 10 km to the west of the FDNPP, their ^129^I activities and ^129^I/^127^I atom ratios were observed to be low, being 0.339–0.422 mBq kg^−1^ and (1.40–1.57) × 10^−8^, respectively^29,30^. However, after the FDNPP accident, the ^129^I activities and ^129^I/^127^I atom ratios in soil increased sharply and presented values of 0.109–160 mBq kg^−1^ and 3.39 × 10^−9^–1.01 × 10^−5^, respectively^8–11,29,30,35,36^. In other words, as shown in Fig. 1, after the FDNPP accident, the maximum values of both ^129^I activities and ^129^I/^127^I atom ratios in Japanese soil samples increased about three orders of magnitude relative to the results of the present study and previous studies as mentioned above. These indicated that the amount of radioiodine released in the FDNPP accident was significant. Owing to the short half-life of ^131^I, it has a high specific radioactivity. Therefore, apart from ^129^I, it is also vital to obtain ^131^I activity values to study the radiation exposure risk from ^131^I to the local population and the environment in this accident.

Due to the short half-life of ^131^I, we could only determine its activities (decay-corrected to collection dates, dry weight) in 22 soil samples, and they were in the range of 1.36–81.3 kBq kg^−1^. Since ^129^I and ^131^I have the same chemical and environmental behaviours, and similar production routes in a nuclear reactor, ^129^I could be an ideal proxy for ^131^I. In soil samples contaminated by the Chernobyl accident, a strong linear correlation was found between ^131^I and ^129^I activities with a ^129^I/^131^I atom ratio of 15.2 ± 4.7 (decay-corrected to April 26, 1986)^7^. Simple slope constants of linearity regressions were also used to reconstruct the ^131^I level and distribution pattern by long-lived ^129^I during the FDNPP accident^8–11^. However, to date, limited ^129^I/^131^I data have been obtained and large errors (most >20%) were observed in previous studies. Four reasons may explain this phenomenon: 1) decreased ^131^I activities by the time of measurement, 2) the time-consuming sample treatment needed for ^129^I analysis by AMS, 3) the limited numbers of AMS systems available, and 4) the light contamination of most regions by the FDNPP accident fallout, but with a large background contribution from multiple sources^8–11^. For example, Miyake *et al*.^10^ reported a relative deviation of 22.1% for ^129^I/^131^I atom ratios in 50 soil samples; Muramatsu *et al*.^11^ even reported a relative deviation of 48.4% for ^129^I/^131^I atom ratios in 82 soil samples. In addition, a higher relative deviation (49.7%) was also observed in the present study. It is vital to compile as many ^129^I/^131^I data as possible to obtain a comprehensive relationship between ^129^I and ^131^I in soil affected by the FDNPP accident fallout and global fallout. However, if more samples with strong background (global fallout) contribution are added, the relative deviation of ^129^I/^131^I atom ratios will increase sharply as mentioned above. Therefore, only using simple slope constants of linearity regressions is not an ideal way to express the relationship between ^129^I and ^131^I, since the region heavily contaminated by the FDNPP accident is restricted to a narrow strip.

**Figure 2:**
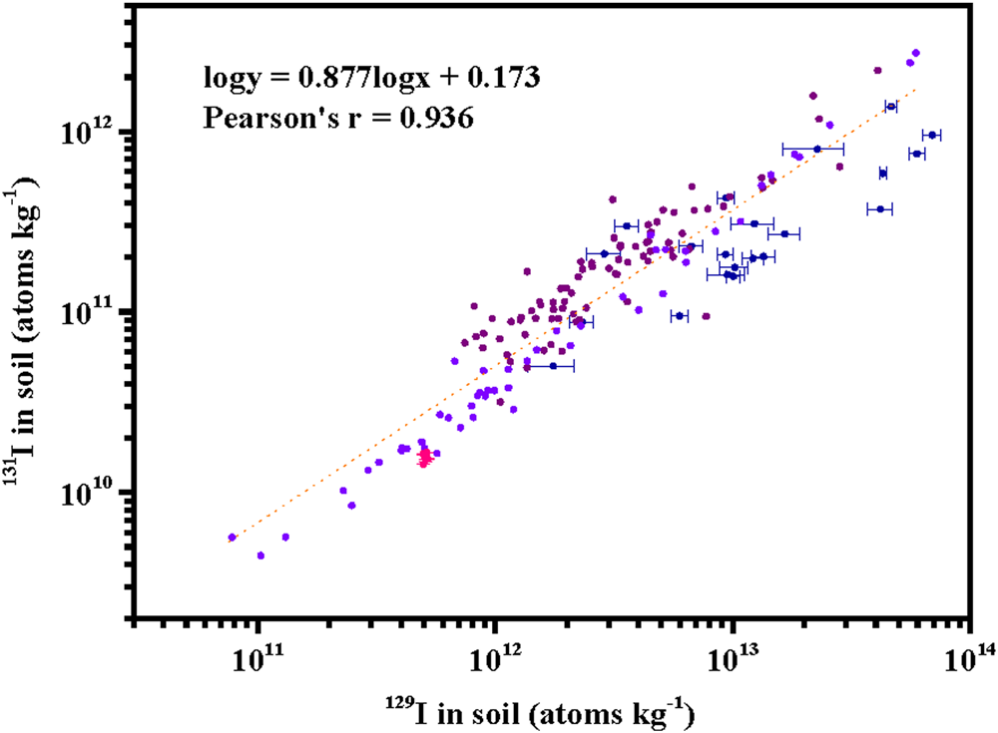
Atom ratio of radioiodine isotopes in Japanese topsoil samples collected after the FDNPP accident. ^131^I activities were decay-corrected to March 11, 2011. The data were from our study (dark blue, errors of 1σ) and previous studies (the data in violet from Miyake *et al*.^10^ and the data in purple from Muramatsu *et al*.^11^ did not show errors, and the data in pink with errors of 1σ were from Fujiwara *et al*.^8^). The dotted line was obtained by linear regression corresponding to the equation in the figure.

We used the available ^129^I and ^131^I data in soil samples, affected by the FDNPP accident fallout and global fallout, in previous studies together with the data in the present study to illustrate a more comprehensive relationship between ^129^I and ^131^I. After a linear regression analysis, the slope of the line indicates the ^129^I/^131^I atom ratio is 26.1 ± 15.5 (decay-corrected to March 11, 2011) and there is no drop in the uncertainties. This indicates again that a simple slope constant of linearity regression cannot explain the relationship between ^129^I and ^131^I comprehensively. After linear regression of all data in Fig. 2 expressed as common logarithms, the equation of logy = 0.877logx + 0.173 (Pearson’s r = 0.936; x, ^129^I concentration (atoms kg^−1^); y, ^131^I concentration (atoms kg^−1^); decay-corrected to March 11, 2011) could be obtained corresponding to the linear regression line. The standard errors for slope and intercept of this equation were low, being 0.030 and 0.33, respectively. The slope <1 in the log-log plot clearly points out systematically varying isotope ratios. Fujiwara *et al*.^8^ reported that the contributions of background ^129^I in five topsoil samples collected in Tsukuba, Japan, about 170 km southwest of the FDNPP, ranged from 38.9% to 41.4%. However, Matsunaka *et al*.^29,30^ observed smaller contributions of background ^129^I in two topsoil samples collected within 10 km to the west of the FDNPP (<5%) due to the heavy contamination from the FDNPP accident. All these indicated the complicated relationships of the sources and contributions for iodine isotopes in the studied soil samples. In addition, Nishihara *et al*.^37^ carried out a model calculation for the radionuclides in the three FDNPP reactors using the ORIGEN2 code, and they estimated significantly distinct ^129^I/^131^I atom ratios at the time of this accident which were 31.4, 21.9, and 20.8 for reactor Units 1, 2, and 3, respectively. Therefore, a larger error in more samples for the ^129^I/^131^I atom ratio may indicate the significant influence by background ^129^I in some soil samples and a complicated situation of radioiodine release from the three reactor units during the FDNPP accident. Anyway, presently, using ^129^I as a proxy for ^131^I offers the best way to estimate the radiation exposure risk from ^131^I to the local population and the environment. Therefore, at present, the equation of logy = 0.877logx + 0.173 (Pearson’s r = 0.936; x, ^129^I concentration (atoms kg^−1^); y, ^131^I concentration (atoms kg^−1^); decay-corrected to March 11, 2011) instead of a simple slope constant of linearity regression with large uncertainty may be a better way to express the relationship between ^129^I and ^131^I in the soil samples in Fukushima Prefecture affected by both global fallout and the FDNPP accident fallout.

**Figure 3:**
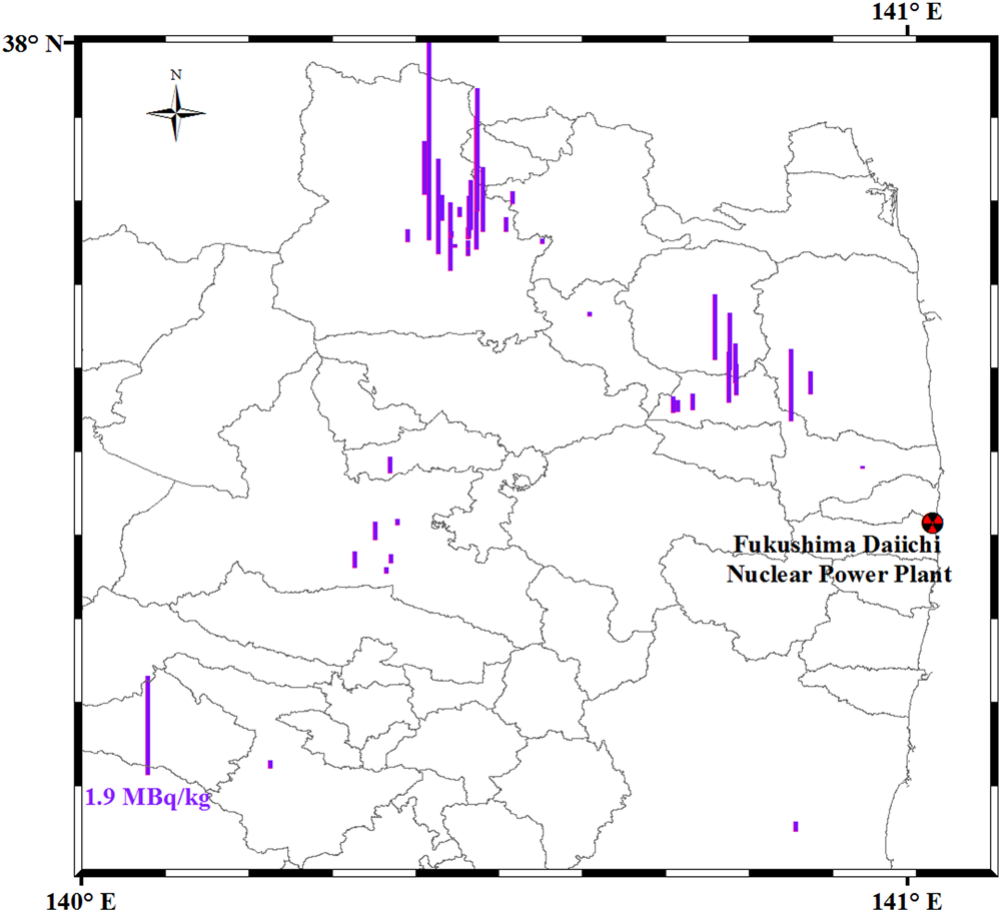
The distribution of ^131^I activity (decay-corrected to March 11, 2011) in the soil samples contaminated by the FDNPP accident fallout and global fallout. This map was prepared with Arc GIS 10.3 software.

As shown in Fig. 3 (decay-corrected to March 11, 2011, to facilitate comparison with other studies), the reconstructed ^131^I activities were 0.023–60.3 kBq kg^−1^ (decay-corrected to collection dates, dry weight); and, higher ^131^I activities were observed in the northwest direction from the FDNPP, in agreement with the observation that the radionuclides were predominately deposited northwest of the facility in a band approximately 40 km in length^11^. It should be noted that only a few samples were collected in the southwest direction from the FDNPP; therefore, in the future more samples from this area are required to show the distribution of ^131^I and correlation between ^129^I and ^131^I more exactly.

**Figure 4:**
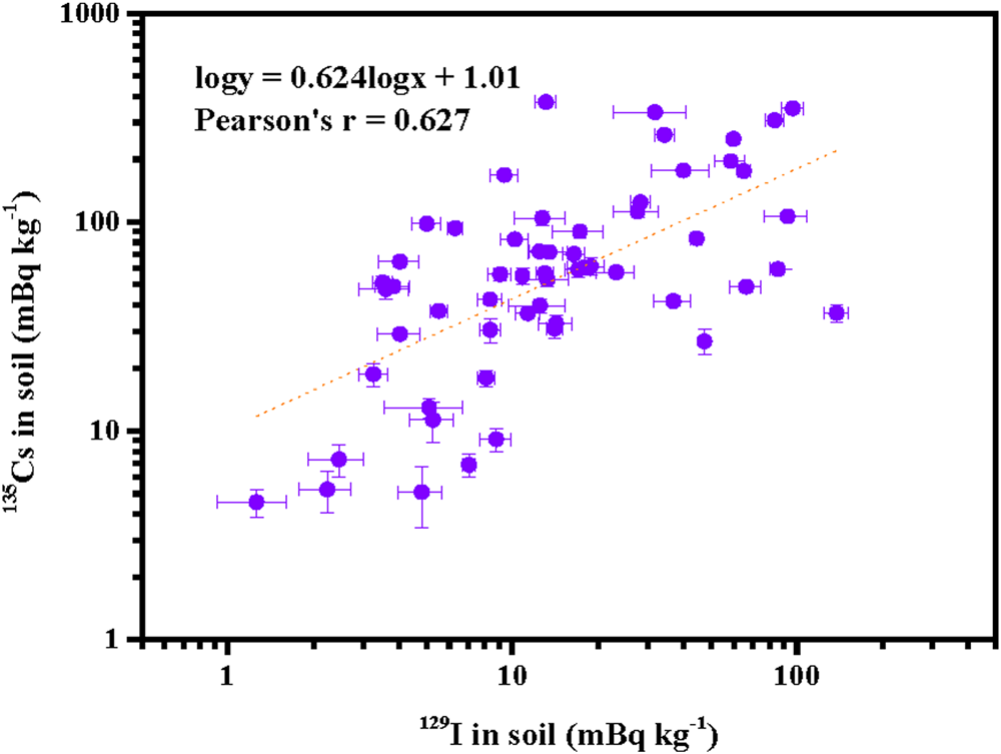
Correlation between ^135^Cs and ^129^I activities in soil samples collected after the FDNPP accident.

After the FDNPP accident, more than 99% of the released activity was due to radionuclides of the elements Kr, Te, I, Xe, and Cs; however, little work had been done on monitoring of radionuclides other than the short-lived ^131^I, ^132^Te, ^134^Cs, ^136^Cs, and ^137^Cs^1^. Radionuclides such as those of less volatile elements (e.g., Pu) and radionuclides with very long half-lives (e.g., ^135^Cs, ^129^I, and some actinides such as ^236^U) have been understudied by comparison^4,5,13,16,21^. Therefore, in the present study, we combined the data for ^135^Cs, ^236^U, ^239^Pu, and ^240^Pu in the same samples with the data of the radioiodine isotopes, trying to see whether ^129^I or ^131^I could trace other radionuclides in soil samples. To the best of our knowledge, this is the first time these long-lived radioisotopes have been simultaneously compared in the same samples. As shown in Fig. 4, ^135^Cs and ^129^I activities had a moderate linear correlation (logy = 0.624logx + 1.01, Pearson’s r = 0.627; x, ^129^I activity (mBq kg^−1^); y, ^135^Cs activity (mBq kg^−1^)). Very recent studies have proved that ^135^Cs/^137^Cs is a potential tracer of radiocesium in environmental science, however, the data of ^135^Cs in soil samples are few in number and no ^135^Cs data in the Japanese environmental samples before the FDNPP accident are available^5^. Although in the course of the FDNPP accident, Matsunaka *et al*.^30^ found that the ^129^I/^137^Cs ratios were varying by several orders of magnitude, we consider it feasible to roughly estimate ^135^Cs activities in soil samples using published ^129^I activities, under the special conditions that significantly fewer numbers of ^135^Cs data in soil are available compared with ^129^I data at present and most fractions of ^135^Cs in the soil are from global fallout rather than the FDNPP accident fallout^5^. In addition, the ^236^U/^129^I, ^239^Pu/^129^I, and ^240^Pu/^129^I activity ratios varied largely from 7.84 × 10^−5^ to 1.23 × 10^−1^, from 0.144 to 72.9, and from 0.102 to 49.7, respectively. ^129^I did not show significant correlations with ^236^U, ^239^Pu, and ^240^Pu. The major reason may be that 1) the releases of ^236^U, ^239^Pu, and ^240^Pu from the FDNPP accident were trace amounts compared with the previous depositions of the radioiodine isotopes; 2) most of the ^236^U, ^239^Pu, and ^240^Pu present in Japanese soil samples are from global fallout^4,5,13,16,21^; 3) ^129^I and ^135^Cs are volatile radionuclides, while ^236^U, ^239^Pu, and ^240^Pu are less volatile radionuclides. Therefore, the differences between ^129^I and ^236^U, ^239^Pu, or ^240^Pu become larger than that with ^135^Cs after release. From the present study, we have confirmed that ^129^I in Japanese soil could roughly track ^135^Cs, rather than ^236^U, ^239^Pu, and ^240^Pu derived from the FDNPP accident fallout and global fallout.

It should be noted that the uncertainty of ICP-QQQ is somewhat larger than that of AMS at present. However, the amount of ^129^I in the unprocessed spent nuclear reactor fuel is 10 times more than that released to the environment and more ^129^I will be produced with the increasing number of nuclear power reactors being built. If most of the spent fuel is going to be reprocessed and ^129^I is released to the environment, it may increase the ratio of ^129^I/^127^I to 10^−3^ ^38^. Then, the ICP-QQQ method will become a more feasible method to investigate ^129^I in the environment with smaller uncertainties.

## Methods

### Soil Sampling

The procedure details for soil sampling and pre-treatment have been described elsewhere^27^. Surface soils (0–5 cm) were collected from 60 sites in Fukushima Prefecture (Fig. 3 and Supplementary Table S1) during four sampling expeditions conducted in 2011, from April 12 to 16, April 26 to 28, June 6 to 10, and June 15 to 16. The collection sites were mainly restricted to the heavily contaminated region where the radioactive plume due to the FDNPP accident was washed out by rainfall. Fukushima Prefecture is divided by mountain ranges into three regions (from west to east) showing large temperature and weather contrasts^21^. On average, annually, central Fukushima receives 1166 mm of precipitation and 189 cm of snow, respectively.

After large particles and plant roots were removed by handpicking, soil samples were transferred into 100-mL polystyrene containers, and then, only the fine fraction of soil particles (diameter below 2 mm) was analyzed.

### Reagents and Materials

Ultrapure grade 25% TMAH (TAMAPURE-AA) was obtained from Tama Chemicals (Kawasaki, Japan). Analytical grade (NH_4_)_2_SO_3_ solution (0.6–1%), 0.1 mol L^−1^ KI solution, CCl_4_, and NaNO_2_ were obtained from Kanto Chemical Co. Inc. (Tokyo, Japan). Single-element standard solutions (1000 mg L^−1^) of Cs, Mo, Cd, In, Li, Mg, Y, Ce, Tl, and Co were also purchased from Kanto Chemical Co. Inc. K_2_S_2_O_8_ was obtained from Wako Pure Chemical Industries, Ltd. (Osaka, Japan). High purity O_2_ (>99.999%) and Ar (>99.999%) were supplied by Taiyo Nippon Sanso Corp. (Tokyo, Japan). IAEA reference material (soil, IAEA-375), NIST standard reference materials (marine sediment, NIST SRM 4357), and GSJ Geochemical reference materials (rock, JB-2 and JB-3; stream sediment, JSd-3) were used for iodine method validation, as shown in Supplementary Tables S2 and S3.

### Analysis of Radionuclides

An Agilent 8800 (ICP-QQQ, Agilent Technologies, Santa Clara, CA, USA), featuring an octopole collision/reaction cell situated between two quadrupole mass filters (first, Q1; second, Q2), was employed for ^129^I/^127^I atom ratio analysis. Pure O_2_ (>99.999%) was introduced into the collision/reaction cell via the No. 4 cell gas line. A high efficiency sample introduction system (APEX-Q, Elemental Scientific, Omaha, NE, USA) equipped with a PFA MicroFlow nebulizer was used as the sample introduction system. The ICP-QQQ was optimized on a daily basis using 1 ng mL^−1^ of standard solution containing Ce, Co, Li, Mg, Tl, and Y in 0.01% (NH_4_)_2_SO_3_. The optimized operation parameters are summarized in Supplementary Table S4. ^133^Cs was measured as internal standard by ICP-QQQ single MS mode for ^127^I concentration measurement. In addition, ^95^Mo, ^111^Cd, and ^115^In were monitored to check the contributions of their polyatomic interferences at *m/z* 129 during the analysis of ^129^I by ICP-QQQ. Activity of ^131^I was determined by γ-ray spectroscopy (ORTEC GEM-40190, Seiko-EG&G, Tokyo, Japan) at an energy of 636 keV from 1000 to 80,000 s^27^. Mixed gamma standard sources with different sample heights were used for efficiency correction; they were obtained from the Japan Radioisotope Association. Because the measurements were started in the middle of May 2011, ^131^I could only be determined in 22 soil samples.

### TMAH Exaction and Purification of Iodine for ICP-QQQ Analysis

The procedures for iodine extraction from solid environmental samples and purification (solvent extraction and back-extraction) for ICP-QQQ analysis are summarized in Supplementary Fig. S1. Six steps are shown here briefly. (1) *10% TMAH extraction*: eighteen of soil samples (1 g) were weighted into 50 mL centrifuge tubes, and then incubated for iodine extraction by 10% TMAH solution (25 mL). Incubation was done in an aluminium block bath (DryThermoUnit DTU–2CN, TAITEC Corp., Koshigaya, Japan) at 90 ^o^C for 2 h. (2) *Iodine release from organic matter*: after centrifugation at 1000 rpm for 5 min (these same parameters for centrifugation were used in the following sections), the aqueous phase was transferred to a new centrifuge tube. A 0.1 mL aliquot of the aqueous phase was taken out, and diluted with 0.01% (NH_4_)_2_SO_3_ to ensure the measured ^127^I concentrations remain within the range of the calibration curve (0.01–5 ng mL^−1^). At the same time, Cs was added to get a final concentration of 5 ng mL^−1^ as internal standard for ICP-QQQ single MS mode analysis since the extracted Cs amount was ignorable compared with the added amount. For the remaining extracted TMAH solution, after adding K_2_S_2_O_8_ (about 0.03 g), the solution was incubated at 60 °C in the aluminium block bath overnight to convert organic iodine to inorganic iodine (iodate, IO_3_^−^). Then, the precipitate was discarded after centrifugation. (3) *IO*_3_^−^ *reduction*: CCl_4_ (5 mL) was added to avoid element iodine release during the following chemical reaction. After adding 1% (NH_4_)_2_SO_3_ (1.5 mL) and then adding 6 M HNO_3_ (9 mL) to adjust pH < 3, IO_3_^−^ was reduced to iodide (I^−^) by (NH_4_)_2_SO_3_ (IO_3_^−^ + 3SO_3_^2−^ → I^−^ + 3SO_4_^2−^). (4) *Organic layer removal*: after mechanical shaking for 2 min and then centrifuging, the organic matter became a thin layer between the TMAH phase and the CCl_4_ phase. The upper TMAH phase and the bottom CCl_4_ phase were transferred to another 50 mL centrifuge tube, and the black organic matter was discarded. (5) *I*_2_ *transformation*: I^−^ was oxidized to elemental iodine (I_2_) by addition of 5% NaNO_2_ (0.4 mL) under acidic conditions (NO_2_^−^ + 2I^−^ + 4 H^+^  → I_2_ + 2NO + 2H_2_O). At the same time, I_2_ was extracted with CCl_4_ (5 mL) by mechanical shaking for 2 min. After centrifugation, the organic phase was separated from the aqueous phase for the next procedure. Elemental iodine was extracted again with CCl_4_ (3 mL). (6) *I* ^−^ *transformation and extraction*: I_2_ in the organic phase was reduced and extracted back into 0.01% (NH_4_)_2_SO_3_ solution (1.5 mL) as I^−^ (I_2_ + H_2_O + SO_3_^2−^ → 2 H^+^ + 2I^−^ + SO_4_^2−^) (back-extraction). Since the shaking during back-extraction was not done strongly, no emulsion appeared in the bottom phase; this helped to increase iodine recovery. Finally, the ^129^I/^127^I atom ratio was analyzed by ICP-QQQ MS/MS mode. For 1 g soil samples, the method detection limits for ^127^I and ^129^I in ICP-QQQ MS-MS mode were 3.80 ng kg^−1^ and 2.62 × 10^–4^ Bq kg^−1^, respectively. This method could measure ^129^I/^127^I > 10^−8^ accurately, as shown in Supplementary Tables S2 and S3.

In addition, the data of ^135^Cs, ^236^U, ^239^Pu and ^240^Pu obtained in our previous studies^5,14,20,21^, were combined with the present data of ^129^I and ^131^I as a means to illustrate the differences and relevance among them in soil samples affected by the FDNPP accident fallout and global fallout, in order to see whether ^129^I can track other radionuclides (^135^Cs, ^236^U, ^239^Pu, and ^240^Pu) derived from the FDNPP accident fallout and global fallout. Details about the analysis of ^135^Cs, ^236^U, ^239^Pu and ^240^Pu can be found in the Supplementary Information.

## Electronic supplementary material

Supplementary Information
