# Supplementary material for: Can 129I track 135Cs, 236U, 239Pu, and 240Pu apart from 131I in soil samples from Fukushima Prefecture, Japan?
Source: Sci Rep. 2017 Nov 13;7:15369. doi: 10.1038/s41598-017-15714-w (PMC5684215; doi:10.1038/s41598-017-15714-w)
Supplement: Supplementary file 1 — Supplementary Information [file 41598_2017_15714_MOESM1_ESM.pdf]

Supplementary Information

Can ^{129}I track ^{135}Cs, ^{236}U, ^{239}Pu, and ^{240}Pu apart from ^{131}I in soil samples from Fukushima Prefecture, Japan?

Guosheng Yang[1,2,3], Hirofumi Tazoe[1] & Masatoshi Yamada[1,*]

[1]Department of Radiation Chemistry, Institute of Radiation Emergency Medicine, Hirosaki University, 66-1 Hon-cho, Hirosaki, Aomori 036-8564, Japan

[2]Division of Nuclear Technology and Applications, Institute of High Energy Physics, Chinese Academy of Sciences

[3]Beijing Engineering Research Center of Radiographic Techniques and Equipment, Beijing 100049, China

Number of pages in Supporting Information Section: 15, including cover

Number of Tables in Supplementary Information Section: 4

Number of Figures in Supplementary Information Section: 3

---

*Corresponding author. Tel.: +81 172 39 5405; Fax: +81 172 39 5405.

myamada@hirosaki-u.ac.jp

Methodology

Analysis of Cs Isotopes. The concentrations of ^{134}Cs and ^{137}Cs were determined by γ-ray spectroscopy (ORTEC GEM-40190, Seiko-EG&G, Tokyo, Japan) at energies of 604 keV and 662 keV, respectively. Mixed gamma standard sources obtained from the Japan Radioisotope Association were employed for efficiency correction. After organic matter decomposition in a muffle oven at 450 ^{o}C for 2 h, leaching with concentrated HNO_3 (20 mL) was performed in PFA jars with lids (Savillex, Eden Prairie, MN, USA) on a hot plate at 180 ^{o}C for 2 h. During acid leaching, for those samples with high organic contents, H_2O_2 (5 mL) was added. After filtration, the sample solutions were adjusted to 4 M HNO_3 for Cs separation. The purification of Cs was conducted as described in Supplementary Fig. S2 following the method of Yang *et al.* [1]. Triple - quadrupole inductively coupled plasma - mass spectrometry (ICP-QQQ, Agilent Technologies, Santa Clara, CA, USA) featuring an octopole collision/reaction cell situated between quadrupole mass filters (first, Q1; second, Q2) was then employed for the analysis of $^{134}Cs/^{137}Cs$ and $^{135}Cs/^{137}Cs$ atom ratios. Finally, ^{135}Cs activity could be obtained by combining the data from γ spectrometry and ICP-QQQ.

Analysis of U Isotopes. The separation procedure for U isotopes was conducted as

described in Supplementary Fig. S3 following the method of Yang *et al.* [2]. Soil samples (about 1 g) were ashed in a muffle oven at 450 ^{o}C for 2 h to decompose organic matter. Total dissolution (HF (10 mL) + HNO_3 (5 mL) + $HClO_4$ (3 mL)) was performed in PFA jars with lids (Savillex) on a hot plate at 180 ^{o}C for 1 d. After filtration, Si was removed by reaction with 46% HF. Then the HF solution was heated to dryness, after which 61 % HNO_3 (5 mL) was added and this acid solution was heated to dryness to remove residual HF. Subsequently, the sample residue was dissolved into 6 M HNO_3 (10 mL), ready for chromatographic purification using DGA resin. Finally, the U eluate was evaporated to near dryness and the wet residue was dissolved into 4% HNO_3 (1.5 mL). An aliquot (20 μL) was taken out and diluted with 4% HNO_3 at a dilution factor of 2000 for ^{238}U concentration measurement by ICP-QQQ single MS mode. The remaining portion was analyzed for ^{236}U/^{238}U atom ratio by ICP-QQQ MS/MS mode.

Analysis of Pu Isotopes. Preparation and purification of Pu isotopes were done based on previous study with a modification from the method of MEXT (Ministry of Education, Culture, Sports, Science and Technology, Japan) [3]. Briefly, soil samples (10 g) were ashed in a muffle oven at 500 ^{o}C for 3 h to decompose organic matter. The ashed samples were digested by heating on a hotplate using a mixture of 10 M HNO_3

and 1 M HF. After filtration, the Pu solution was loaded on a column and purified by anion-exchange chromatography (Dowex 1X8) and then electrodeposited onto a stainless steel disc. The activities of ^{238}Pu and $^{239+240}$Pu were measured with an α-spectrometer. After that, Pu on the stainless steel disc was extracted with a mixture of 10 M HNO_3 and 1 M HF. Plutonium isotopes in the extracted solution were further purified by anion-exchange chromatography. After loading the sample solution on the first Dowex 1X8 column (6mL), sequential elution of U, Th and Pu was conducted using 8 M HNO_3 (120 mL), 10 M HCl (150 mL), and NH_4I-HCl solution (100 mL), respectively. After adding HNO_3 (5 mL) to the final eluate, the obtained Pu fraction was heated to dryness, and the residue was dissolved into 4 M acetic acid (10 mL). This solution was then loaded onto the second Dowex 1X8 column (2 mL) and 4 M acetic acid (20 mL) was used to rinse the column. All of the eluted solution was collected (30 mL) for Pu analysis. The collected acetic acid solution was heated to dryness and the residue was dissolved into 4% HNO_3 (10 mL) for ^{239}Pu and ^{240}Pu analysis using an APEX-Q/SF-ICP-MS (ELEMENT 2, Thermo Fisher Scientific, Bremen, Germany). The chemical yields of Pu were determined using a ^{242}Pu yield tracer with negligible quantities of ^{239}Pu and ^{240}Pu. ^{242}Pu/^{239}Pu and ^{242}Pu/^{240}Pu were used to create an external calibration to calculate the activities of Pu isotopes.

Table S1. Iodine isotopes in Japanese soil contaminated by the FDNPP accident fallout and global fallout.

Sample	Land use	Collection date	Longitude (°N)	Latitude (°E)	^{127}I (ng g^{-1})	$^{129}I/^{127}I$ atom ratio ($\times 10^{-6}$)	^{129}I (mBq kg^{-1})	^{131}I (kBq kg^{-1})
S12	Uncultivated land	2011/4/12	37.5744	140.8822	536±30	3.68±0.21	13.1±1.1	428±71
S13	Uncultivated land	2011/4/12	37.5744	140.8822	819±14	1.72±0.19	9.37±1.04	231±45
S14	Uncultivated land	2011/4/12	37.5419	140.8594	1911±19	5.12±0.26	65.0±3.3	1373±59
S15	Uncultivated land	2011/4/13	37.5733	140.7931	16269±86	0.554±0.018	59.9±2.0	586±62
S16	Home ground	2011/4/13	37.6036	140.7844	20041±170	0.300±0.070	39.9±9.3	
S19	Paddy field	2011/4/13	37.5538	140.7216	1852±27	0.680±0.057	8.36±0.71	95±19
S20	Paddy field	2011/4/13	37.5538	140.7216	2084±10	0.941±0.068	13.0±0.9	207±23
S23	Paddy field	2011/4/13	37.5538	140.7216	2106±11	1.22±0.13	17.0±1.8	198±26
S27	Paddy field	2011/4/13	37.5538	140.7216	2203±13	0.908±0.164	13.3±2.4	161±22
S29	Green house without cover	2011/4/13	37.5538	140.7216	16000±117	0.132±0.008	14.1±0.9	157±20
S35	Green house with cover	2011/4/14	37.5538	140.7216	9618±42	0.022±0.002	1.37±0.13	
S36	Paddy field	2011/4/14	37.5538	140.7216	2646±29	0.055±0.012	0.962±0.203	
S37	Paddy field	2011/4/14	37.5538	140.7216	2297±40	0.161±0.035	2.46±0.54	50±10
S39	Home ground	2011/4/14	37.5561	140.7398	8245±30	0.344±0.039	18.9±2.1	202±24
S40	Upland field	2011/4/14	37.5519	140.7159	21348±134	0.029±0.009	4.12±1.22	
S42	Upland field	2011/4/14	37.5519	140.7159	3823±32	0.680±0.136	17.3±3.5	308±33
S44	Pasture	2011/4/14	37.5519	140.7159	23617±119	0.148±0.022	23.2±3.5	269±29
S45	Home ground	2011/4/15	37.5561	140.7398	21164±144	0.197±0.023	5.01±0.58	299±35
S46	Home ground	2011/4/15	37.5651	140.7839	1672±78	2.86±0.81	31.7±9.1	797±64
S47	Pasture	2011/4/15	37.5651	140.7839	2260±27	1.95±0.24	58.5±7.1	371±50
S48	Pasture	2011/4/15	37.5651	140.7839	13460±95	1.08±0.09	96.7±8.4	954±77
S49	Paddy field	2011/4/15	37.5881	140.7919	4509±45	2.79±0.21	83.5±6.3	752±64
S54	Home ground	2011/4/16	37.6161	140.7672				1243±113
S55	Park ground	2011/4/26	37.1221	140.2285	9277±67	0.083±0.025	5.10±1.56	
S56	Playground	2011/4/26	37.0451	140.8649	1230±17	0.861±0.043	7.04±0.36	
S57	Parking lot	2011/4/27	37.3706	140.3752	2407±25	0.345±0.023	5.51±0.38	
S58	Park	2011/4/27	37.7628	140.4685	3186±27	0.673±0.089	14.2±1.9	176±18

S59	Road side	2011/4/27	37.7628	140.4684	121±17	5.00±0.432	4.03±0.65	210±23
S60	Park	2011/4/27	37.4847	140.9451	2749±11	0.069±0.019	1.26±0.34	
S61	Park	2011/4/28	37.6686	140.6148	187±3	2.62±0.30	3.25±0.38	88±15
S62	Road side	2011/6/6	37.7892	140.4583	174±2	5.44±0.33	6.30±0.40	
S63	Road side	2011/6/6	37.7741	140.4710	3366±35	1.53±0.12	34.2±2.7	
S64	Road side	2011/6/6	37.7423	140.4676	1207±61	0.600±0.103	4.81±0.86	
S65	Road side	2011/6/6	37.7508	140.4679	1675±82	0.473±0.079	5.26±0.91	
S66	Road side	2011/6/7	37.7637	140.4686	1688±64	1.20±0.19	13.5±2.1	
S67	Road side	2011/6/7	37.7656	140.4694	1637±56	2.59±0.19	28.2±2.3	
S68	Road side	2011/6/8	37.8052	140.5221	1020±38	1.23±0.11	8.36±0.81	
S69	Road side	2011/6/8	37.7708	140.5144	737±35	2.08±0.21	10.2±1.1	
S70	Road side	2011/6/8	37.7563	140.5579	134±7	4.02±0.77	3.59±0.71	
S71	Road side	2011/6/8	37.7501	140.4782	3420±15	4.07±0.69	92.6±15.7	
S72	Road side	2011/6/9	37.7246	140.4467	8794±10	0.809±0.020	47.3±1.1	
S73	Road side	2011/6/9	37.7957	140.4789	8447±26	1.53±0.09	85.6±5.3	
S74	Road side	2011/6/9	37.7719	140.4857	1832±15	1.05±0.21	12.7±2.6	
S75	Road side	2011/6/9	37.7719	140.4857	1309±8	1.44±0.32	12.5±2.8	
S76	Road side	2011/6/9	37.7719	140.4857	2625±2	0.520±0.049	9.06±0.85	
S77	Road side	2011/6/9	37.7719	140.4857	3283±10	1.26±0.23	27.6±5.0	
S78	Road side	2011/6/9	37.7719	140.4857	2771±25	0.477±0.058	8.78±1.06	
S79	Road side	2011/6/9	37.7719	140.4857	6499±19	1.03±0.05	44.4±2.2	
S80	Road side	2011/6/9	37.7719	140.4857	1873±5	1.32±0.12	16.5±1.5	
S81	Road side	2011/6/9	37.7643	140.4483	464±7	1.24±0.17	3.82±0.53	
S82	Road side	2011/6/9	37.7849	140.4368	1900±23	1.40±0.16	17.7±2.1	
S83	Road side	2011/6/9	37.8166	140.4149	698±10	7.93±1.17	36.8±5.4	
S84	Road side	2011/6/9	37.7614	140.4207	6092±7	3.41±0.34	138±14	
S85	Road side	2011/6/9	37.7441	140.4322	8706±43	1.15±0.14	66.3±8.2	
S86	Road side	2011/6/9	37.7518	140.4524	432±10	0.780±0.161E	2.24±0.47	
S87	Road side	2011/6/10	37.7591	140.3945	1677±38	0.725±0.032	8.09±0.54	
S88	Road side	2011/6/15	37.3651	140.3314	1683±145	0.974±0.074	10.8±0.2	
S89	Parking lot	2011/6/15	37.3575	140.3686	2316±146	0.261±0.028	4.03±0.69	
S90	Park	2011/6/15	37.4164	140.3831	196±10	2.70±0.23	3.51±0.27	
S91	Park	2011/6/15	37.4787	140.3732	1635±101	1.05±0.076	11.4±1.1	
S92	Park	2011/6/16	37.3980	140.3560	3327±6	0.564±0.044	12.5±1.0	

Table S2. ^{127}I concentrations in Japanese standard reference materials (SRMs). This table is cited from our previous study [4].

SRM	Type	Information value(ng g^{-1})	Reference	Our study (ng g^{-1}) (n=3)[4]
JB-2	Rock	49	5	49.2±1.7
JB-3	Rock	28	5	25.6±1.4
JSd-3	Stream sediment	4250±390(n=4)	6	4598±119
		4470±500 (n=3)	6	
		4200±100 (n=8)	7	
		4230±330(n=3)	8	

All errors in this table are shown as 1σ.

Table S3. ^{129}I activity, ^{127}I concentration, and $^{129}I/^{127}I$ atom ratio in the IAEA-375 soil and NIST SRM 4357 marine sediment reference materials. This table is cited from our previous study [4].

$^{129}I/^{127}I$ atom ratio	^{129}I(mBq kg^{-1})	^{127}I(mg kg^{-1})	Analytical methods	n	References
**IAEA-375**					
	1.3–2.1		AMS(2), ID-MS(1), NAA(8)[a]		9
	1.59±0.04		AMS	7	10
$(1.72±0.08)×10^{-7}$	1.64±0.13	1.47±0.08	AMS and ICP-MS	4	11
$(1.69±0.15)×10^{-7}$	1.7±0.3		ICP-QQQ	5	12
$(1.89±0.05)×10^{-7}$	1.60±0.04		AMS	10	13
$(1.61±0.04)×10^{-7}$	1.80±0.03		TIMS	2	13
$(1.80±0.06)×10^{-7}$	1.77±0.07	1.48±0.04	ICP-QQQ	3	4
**NIST SRM 4357**					
$(4.88±0.95)×10^{-8}$	1.14±0.11	3.52±0.76	AMS	12	13
$(4.16±0.19)×10^{-8}$	1.05±0.03	3.80±0.21	TIMS	2	13
$(5.03±1.02)×10^{-8}$	1.32±0.28	3.95±0.11	ICP-QQQ	3	4

AMS, accelerator mass spectrometry; ID-MS, isotopic dilution mass spectrometry; NAA, neutron activation analysis; ICP-QQQ, triple-quadrupole inductively coupled plasma-mass spectrometry; ICP-MS, inductively coupled plasma-mass spectrometry; TIMS, thermal ionization mass spectrometry. All errors in this table are shown as 1σ.

[a]Numbers in parenthesis mean the number of laboratories using corresponding analytical method.

Table S4. Optimized analytical parameters of triple - quadrupole inductively coupled plasma - mass spectrometry (ICP-QQQ). This Table is cited from our previous study [4].

**Plasma**	
RF power	1550 W
RF matching	1.70 V
Sampling position	10.0 mm
Carrier gas	0.88 L min^{-1}
Nebulizer pump	0.10 rps
Makeup gas flow rate	0.30 L min^{-1}
**Lens**	
Extraction lens 1	0.0 V
Extraction lens 2	-140.0 V
Omega bias	-75 V
Omega lens	9.0V
Q1 entrance	1 V
Q1 exit	1 V
Cell focus	2.0 V
Cell entrance	-50 V
Cell exit	-60 V
Deflection	4.0 V
Plate bias	-70 V
**No. 4 cell line**	
O_2 flow rate	0.5 mL min^{-1}
**Integration time**	
m/z (Q1→Q2): Mo 95 → 95, Cd 111→ 111, In 115→ 115	3s
m/z (Q1→Q2): 127 → 127, 129→ 129	4.98 s and 9 s, respectively
**Signal intensity**	
$^{127}I^+$ for 1 ng mL^{-1} iodine standard solution	~ 6.0×10^4 cps

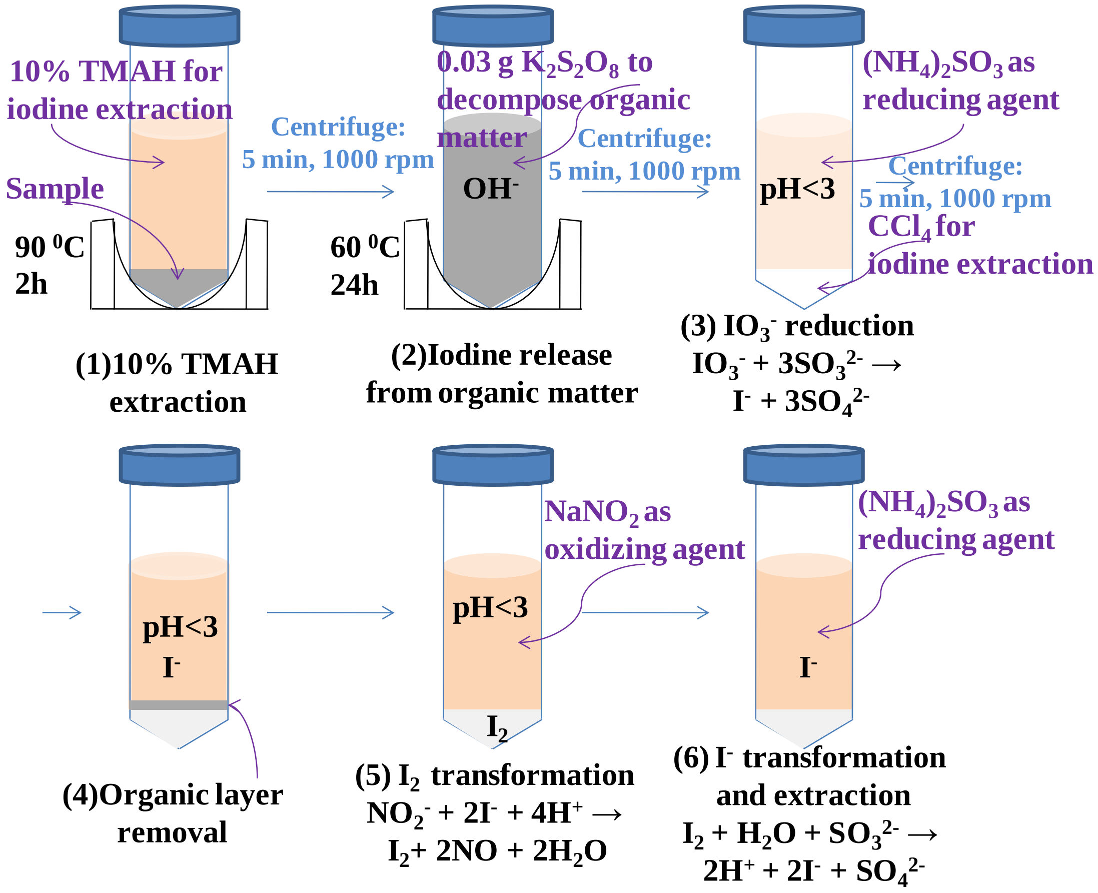

Figure S1. Schematic diagram of procedures for iodine extraction from soil samples and purification for ICP-QQQ analysis. This figure is cited from our previous study [4].

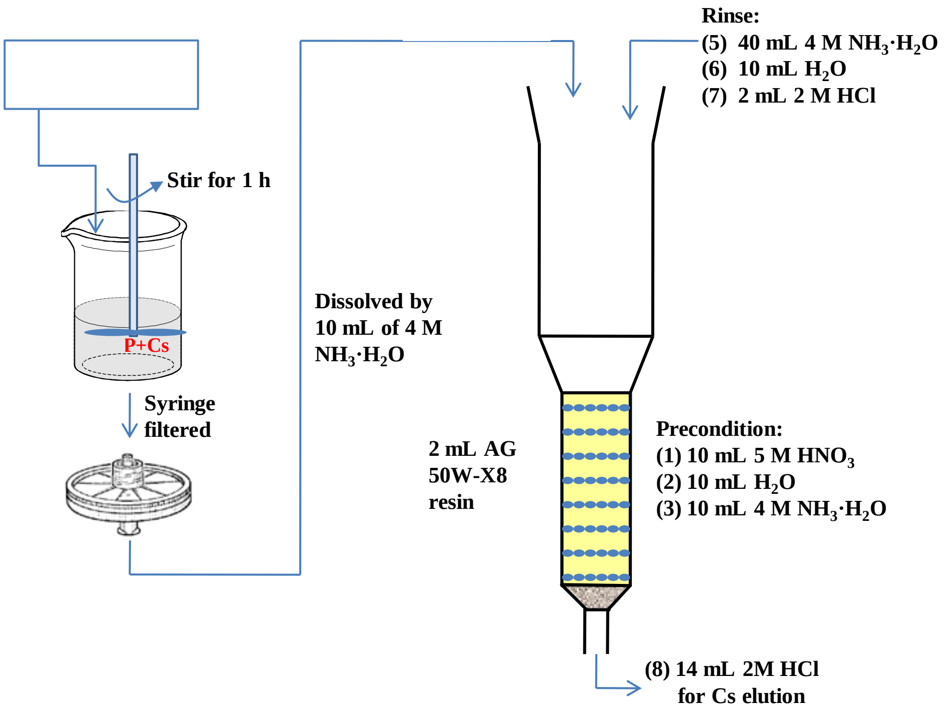

Figure S2. Summary of chemical separation procedure for ^{135}Cs and ^{137}Cs that includes the ammonium molybdophosphate (AMP) selective adsorption and cation exchange chromatography step. The chemical separation procedure is followed by ICP-QQQ MS/MS mode analysis. This figure is cited from our previous study [1].

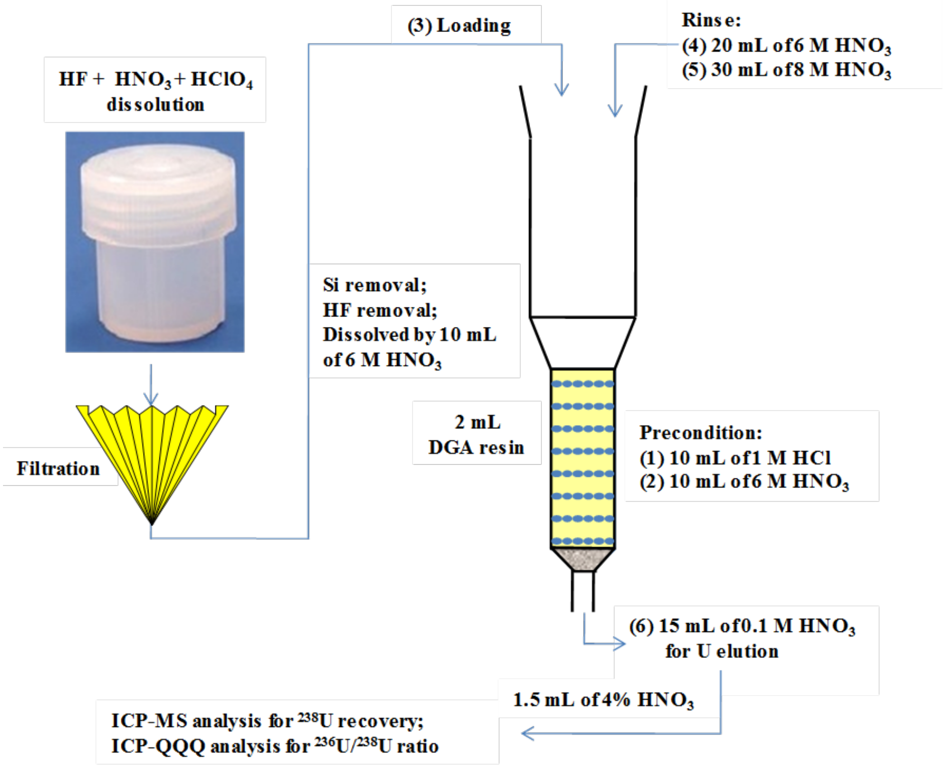

Figure S3. The separation procedure for ^{236}U, incorporating HF + HNO_3 + $HClO_4$ total dissolution and DGA single-column chromatography. The chemical separation procedure is followed by ICP-QQQ analysis. This figure is cited from our previous study [2].

References

1. Yang, G., Tazoe, H. & Yamada, M. Rapid determination of ^{135}Cs and precise $^{135}Cs/^{137}Cs$ atomic ratio in environmental samples by single-column chromatography coupled to triple-quadrupole inductively coupled plasma-mass spectrometry. *Anal. Chim. Acta* **908**, 177−184 (2016).

2. Yang, G., Tazoe, H. & Yamada, M. Determination of ^{236}U in environmental samples by single extraction chromatography coupled to triple-quadrupole inductively coupled plasma-mass. *Anal. Chim. Acta* **944**, 44–50 (2016).

3. Yang, G., Tazoe, H. & Yamada, M. Isotopic compositions of ^{236}U, ^{239}Pu, and ^{240}Pu in soil contaminated by the Fukushima Daiichi Nuclear Power Plant accident. *Sci. Rep*. Manuscript accepted for publication (2017).

4. Yang, G., Tazoe, H. & Yamada, M. Rapid and high throughput determination of ^{129}I activity and $^{129}I/^{127}I$ atom ratio in environmental samples by TMAH extraction combined with triple - quadrupole inductively coupled plasma - mass spectrometry. Manuscript submitted for publication.

5. GSJ Geochemical Reference Samples Data Base. https://gbank.gsj.jp/geostandards/welcome.html.

6. Muramatsu, Y. & Wedepohl, K. H. The distribution of iodine in the earth's crust.

Chem. Geol. **147**, 201–216 (1998).

7. Chai, J. Y. & Muramatsu,Y. Determination of bromine and iodine in twenty-three geochemical reference materials by ICP-MS. *Geostand. Geoanal. Res*. **31**, 143−150 (2007).

8. Sekimoto, S. & Ebihara, M. Accurate determination of chlorine, bromine, and iodine in sedimentary rock reference samples by radiochemical neutron activation analysis and a detailed comparison with Inductively Coupled Plasma Mass Spectrometry literature data. *Anal. Chem*. **85**, 6336−6341 (2013).

9. Strachnov, V., Larosa, J., Dekner, R., Zeisler, R. & Fajgelj, A. Report on the intercomparison run IAEA-375: determination of radionuclides in soil sample IAEA-375. (1996).

10. Jiang, S. S. *et al*. New measurement of ^{129}I in IAEA-375 reference material. *J. Radioanal. Nucl. Chem*. **264**, 549–553 (2005).

11. Muramatsu, Y., Takada, Y., Matsuzaki, H. & Yoshida, S. AMS analysis of ^{129}I in Japanese soil samples collected from background areas far from nuclear facilities. *Quat. Geochronol*. **3**, 291−297 (2008).

12. Ohno, T. *et al*. Determination of ultratrace ^{129}I in soil samples by Triple Quadrupole ICP-MS and its application to Fukushima soil samples. *J. Anal. At. Spectrom*. **28**,

1283–1287 (2013).

13. Olson, J. *et al*. A comparative study of ^{129}I content in environmental standard materials IAEA-375, NIST SRM 4354 and NIST SRM 4357 by Thermal Ionization Mass Spectrometry and Accelerator Mass Spectrometry. *Appl. Radiat. Isot*. http://dx.doi.org/10.1016/j.apradiso.2017.01.028 (2017).
